# Carnivore and Ketogenic-like Diets: Proposed Alternatives for Mitigating and Treating Pediatric Obesity

**DOI:** 10.7759/cureus.83461

**Published:** 2025-05-04

**Authors:** Halee Lair, Holly Haywood, Elena Arcaroli, Braeden Koechle, Kaan Sevgi, Anna K Potter

**Affiliations:** 1 Foundational Science, Nova Southeastern University Dr. Kiran C. Patel College of Osteopathic Medicine, Clearwater, USA; 2 Family Medicine, Nova Southeastern University Dr. Kiran C. Patel College of Osteopathic Medicine, Clearwater, USA; 3 School of Medicine, Kansas City University College of Osteopathic Medicine, Kansas City, USA

**Keywords:** alternative treatments, carnivore diet, diabetes, ketogenic-like diet, obesity, pediatrics

## Abstract

The etiology of the rapidly evolving prevalence of pediatric obesity and diabetes is largely, if not entirely, due to the accessibility and affordability of whole foods and a lack of physical activity. Another likely large factor is health literacy. There is a lack of understanding that overconsumption of highly processed foods and a lack of exercise can lead to diseases even in childhood. Although the etiology of pediatric obesity is vastly multifactorial, diet is a crucial contributing factor, and this is the foundation of our research. This study investigates and compares the proposed efficacy of utilizing carnivore or ketogenic-like diets in treating pediatric obesity and other comorbidities. Current standard guidelines include recommending diets low in protein and high in vegetables, whereas participants implementing our proposed diets would prioritize protein intake. Although there are limited long-term data regarding carnivore or ketogenic-like diets, our research suggests that even short-term application of such a lifestyle will treat and likely prevent many cases of pediatric obesity. Regular physical activity is also encouraged to maximize the benefits of these diets, along with maintaining a mindful diet after the eventual cessation of these treatment diets. These diets have been shown to treat many conditions and show great promise in being realistic alternatives and preventative mechanisms to combat the epidemic of pediatric obesity.

## Introduction and background

Pediatric obesity, an epidemic across the world, significantly influences the immediate and long-term health of patients. The current guidelines for treating and preventing childhood obesity include limiting protein, increasing vegetables, restricting animal-derived products, and increasing physical activity [1]. At first glance, these preventive strategies appear valuable, yet they are deemed inadequate. The risks of severe pediatric obesity are immense and include, but are not limited to, type 2 diabetes mellitus (T2DM), cancer, and cardiovascular morbidity [2]. The sequelae resulting from T2DM in children can be significant and at times life-threatening. Some common presenting symptoms in children with T2DM are polydipsia, polyuria, and weight loss [3]. To confirm the diagnosis serologically, blood glucose and HbA1c levels can be measured. If left unmanaged, T2DM can lead to several complications. In acute cases, untreated patients with diabetes may experience hypoglycemic episodes and more serious, life-threatening diabetic ketoacidosis. Long-term sequelae of poorly managed T2DM include retinopathy, nephropathy, neuropathy, hypertension, and hyperlipidemia [3].

Proper surveillance and screening in childhood can lead to an early diagnosis, which can help mitigate severe complications. Although highly individualized for each patient, the goals of treating T2DM are relatively the same in children. Quantitatively, patients can aim to normalize their HbA1c level. Other goals of treatment include avoiding future complications as well as the adoption of adequate physical activity and proper eating habits [1]. If lifestyle modifications are insufficient at managing the child’s diabetes, insulin therapy is the definitive treatment. Though obesity is not the only etiology of T2DM in children, it is a leading cause [2]. Lifestyle modifications, specifically diet and physical activity, should be prioritized in the treatment of pediatric obesity. The diet clinically recommended for the treatment of obesity should theoretically also help in the treatment of pediatric T2DM. However, studies challenge the current guidelines and suggest that alternative dietary modifications would be more efficacious. The ketogenic and carnivore diets, although primarily implemented for other morbidities, have demonstrated effects that would help manage obesity and diabetes in children.

The ketogenic diet, more commonly known as the "Keto Diet," is characterized by a low-calorie diet that is high in fat and significantly low in carbohydrates [4]. This results in the patient living in a ketotic state, which can help manage many chronic illnesses, including epilepsy and hyperlipidemia. It has even been shown to decrease the risk of cancer [5]. Some research has indicated that these dietary modifications have demonstrated benefit in T2DM patients suffering from obesity, as circulating glucose decreases and insulin sensitivity increases in ketotic states [4]. The carnivore diet, although derived from our pre-human ancestors, has only recently gained popularity in modern society. Although this is a type of ketogenic diet, the foundation of the carnivore diet is built on eliminating foods from plant sources and primarily consuming those sourced from animals [6]. The ideology of this diet relies on the principle that our ancestors chronically consumed a low-carbohydrate, high-protein diet, which led to the positive selection of intrinsic insulin resistance as a survival and reproductive advantage [7]. This insulin resistance in a typical modern diet contributes to the current obesity prevalence. The main characteristics of the diet recommended for pediatric obesity, the ketogenic diet, and the carnivore diet are summarized in Table 1.

**Table 1:** Macronutrient comparison of the different diets Comparison between the carnivore and ketogenic diets based on the recommended levels of carbohydrates, proteins, healthy fats, and plant sources for each diet.  A recommended level of nutrients as an alternative diet-based treatment for pediatric obesity is also included [1,4,7].

	Carbohydrates	Protein	Healthy fats	Plant sources
Carnivore diet	Very low	High	High	No
Ketogenic diet	Very low	Moderate	High	Yes
Recommended diet	Moderate	Low	Moderate	Unspecified

While substantial evidence supports the effectiveness of the ketogenic diet in managing T2D and obesity in adults, there is limited data on its use in pediatric patients with these conditions. This diet has been successfully implemented in epileptic children, which has provided research on side effects and complications. These have been minimal when the diet was implemented appropriately [8]. Studies suggest that patients can safely follow a ketogenic diet for a maximum of six to 12 months [5]. One of the recommendations in the current guidelines for treating childhood obesity is to limit protein intake, but the carnivore diet challenges that idea and suggests that ingesting protein primarily can lead to weight loss by catering to the intrinsic insulin resistance. It is imperative to find a solution quickly for this impending epidemic, since studies demonstrate that insulin producing cells deteriorate faster in children with T2DM than in adults [9]. The objective of this study is to analyze the available literature and explore the carnivore diet as a preventive and therapeutic agent in the treatment of childhood obesity and diabetes.

Methods

A search of the literature published from January 1, 2000, to December 1, 2023, was performed in December 2023 using four databases (PubMed, EMBASE, Ovid MEDLINE, and Web of Science). The searches were conducted using the following search terms: 1) “type 2 diabetes” AND “carnivore diet” AND “adult” OR “kids” OR “adolescents”, 2) “ketogenic diet” AND “children” AND “benefits” OR “long term effects” OR “side effects”, 3) “carnivore diet” AND “essential nutrients” AND “development”. The articles selected for this study were based on primary research articles published since 2000 with a majority of them from the last five years. Articles included data pertaining to carnivore and ketogenic diets and the associated short- and long-term effects, as well as data suggesting or refuting the potential benefits of the aforementioned diets, which may be used in the management of pediatric obesity. No review articles were included in this review. No statistical analysis was conducted, and a Preferred Reporting Items for Systematic Reviews and Meta-Analyses (PRISMA) chart was not used, as it is only applied to scoping reviews, unlike this article.

## Review

Effects of the carnivore diet on obesity, metabolism, and comorbidities

While there is a general agreement that preventing and treating childhood obesity and T2DM requires a multifaceted approach, including the optimization of nutritional behavior, physical activity, and educating children and their families, the components of the recommended diets differ. One study summarized the internationally available strategies and guidelines, which recommend limiting animal-derived products and promoting plant-based foods for early/middle school years and adolescents and deemed them insufficient in treating and preventing childhood obesity [1]. This inadequacy in guidelines necessitates further investigation and trials to control this epidemic.

In the adult population, ketogenic diets have been implemented to control T2DM, likely due to weight loss caused by the diet. There are multiple theories as to why a ketogenic diet leads to weight loss, and most hinge on the idea that the primary energy source becomes ketones or protein [5]. Compared to carbohydrates, it is more difficult for the body to use protein as an energy source, and this results in weight loss [5]. It is also suggested that a diet low in carbohydrates causes increased lipolysis, resulting in fat loss and increased gluconeogenesis, which is considered an expensive metabolic process [4]. Other theories claim that weight loss is not due to the energy source, but rather that satiety is greater in ketotic patients and those who eat higher levels of protein [5]. Although a ketogenic diet is extremely beneficial for weight loss and, consequently, can help to manage comorbidities associated with obesity, it also helps decrease the risk of cancer and both metabolic and neurological disorders [5]. This is achieved by a lower serum ratio of insulin-like growth factor (IGF)/IGF-binding protein 3 and histone deacetylase (HDAC) inhibition found in ketotic states [5].

The carnivore diet is a type of ketogenic diet, and it can be appropriately presumed that both diets offer comparable benefits. The carnivore diet implies a much higher protein intake compared to a standard ketogenic diet, but in cases of childhood obesity and diabetes, a higher protein intake may be advantageous. This dietary approach is based on the hypothesis that our ancestors evolved insulin resistance as an adaptive advantage during periods of low plant availability and high reliance on animal-based foods [7]. While insulin-sensitive individuals may struggle with low-carbohydrate, high-protein diets, those with insulin resistance or a genetic predisposition to obesity often tolerate such diets well [7]. Generally, carbohydrates have been progressively replacing protein in most Western diets, and this has led to an increase in the glycemic index (GI), insulin resistance, and hyperinsulinemia [10]. The GI system measures how much and how quickly the carbohydrate-containing foods raise blood glucose levels after consumption. Foods are scored on a scale from 0 to 100, with pure glucose given the highest value of 100. Low-GI foods (55 or less) are digested and absorbed more slowly, leading to a gradual rise in blood sugar, while high-GI foods (70 or above) cause a rapid rise in blood sugar levels. Maintaining a balance in the Gl of your diet is important for energy stability and metabolic health. Consuming too many high-GI foods can result in blood sugar surges followed by crashes, potentially leading to insulin resistance and an increased risk of diabetes over time. Conversely, consuming primarily low-GI foods can stabilize blood sugar levels but may not provide enough energy during intense physical activity or recovery. While the GI of foods does not differ between adults and children, the latter often have higher metabolic needs and can tolerate higher GI foods, particularly during pubertal growth and after intense physical activity [11]. In addition to the higher GI seen in the recent diets, the decline in the quality of the carbohydrates being produced also supports the trend of increased hyperinsulinemia [10]. When the pancreas is unable to produce enough insulin, glucose intolerance and T2DM develop. In a dietary environment with high carbohydrates and low protein, insulin sensitivity would be a weakness. Therefore, the adaptation towards insulin resistance has become more prevalent [10]. An increase in protein intake and a reduction in glycemic index, as demonstrated in ketogenic, and more specifically, carnivore diets, have been associated with improved weight loss and fewer adverse effects [5].

Neurological effects of the ketogenic diet

Long-term implementation of a ketogenic diet improves gut microbiota by allowing for the proliferation of beneficial bacteria and reducing levels of pro-inflammatory bacteria [12]. Soon after initiation of a ketogenic diet, lethargy is a common complaint, but this seems to resolve relatively quickly and subjects often report improved mood, and when implemented long-term, overall quality of life improvement with few side effects [5]. Studies also show that higher levels of ketones protect the brain from cognitive impairment that can be seen in overweight and obese patients [5]. In a study on rats, elevated levels of excitatory neurotransmitters such as glutamate and glutamine were found in the brain, suggesting that the ketogenic diet provides more energy to the brain than the standard diet [13]. Additionally, metabolites such as glutathione, N-acetyl acetate and glutamate, which are associated with neuroprotective glial cells like astrocytes, were also shown to be increased. The ketogenic diet also led to structural changes in the brain, including increased volume in the pons, and alterations in the striatum which are linked to increased myelinated fibers and improved connectivity between the striatum and other regions of the brain like the hippocampal formation and the fornix [13].

Discussion

**Figure 1:**
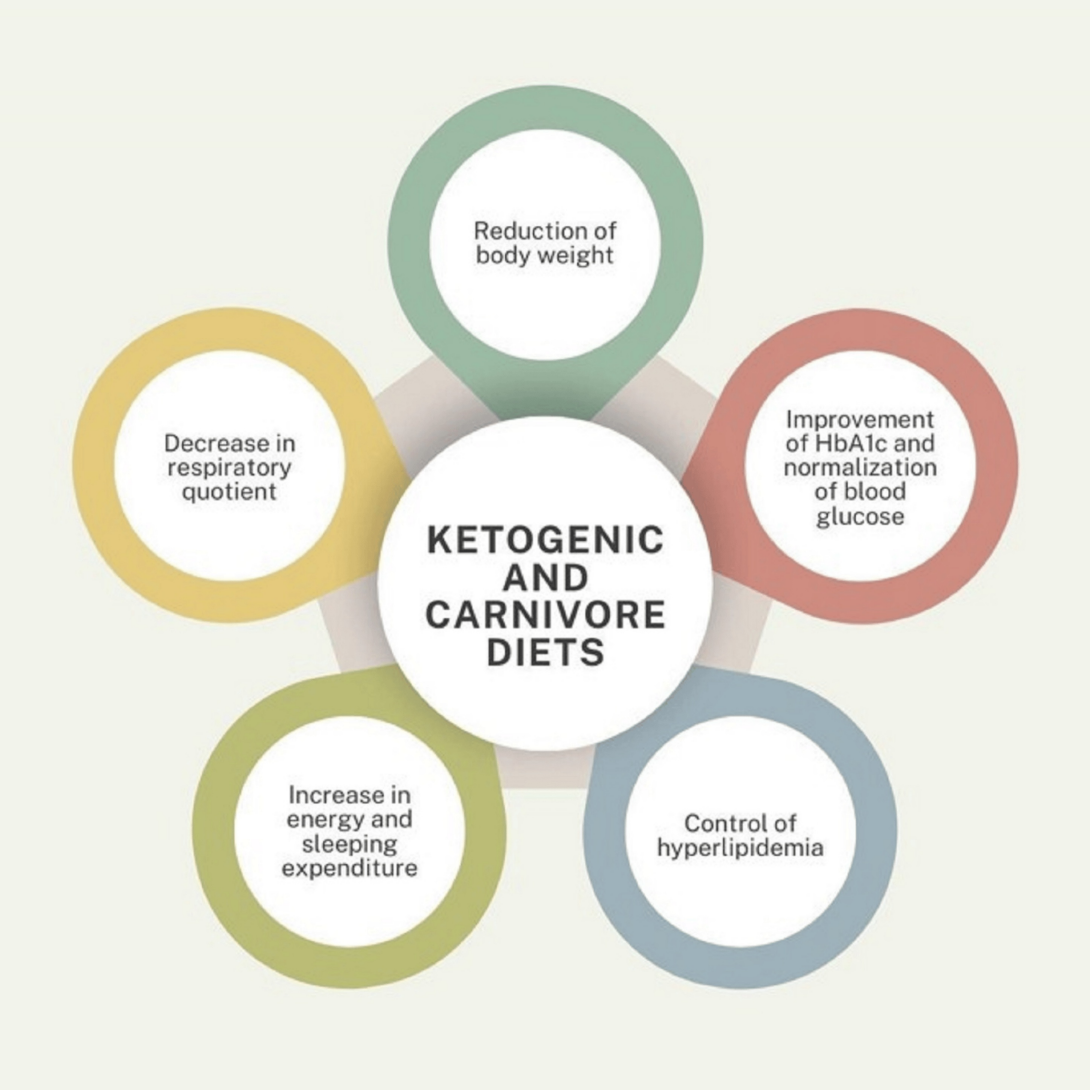
Benefits of ketogenic and carnivore diets Diagram of the overall benefits of the carnivore and ketogenic diets, including improvement of HbA1c and blood glucose levels, control of hyperlipidemia, and over all increased health benefits [5,14]. Graphic credit: created by Elena Arcaroli on Canva (Canva Pty Ltd., Syndney, Australia)

The reduction in body weight, improvement in the lipid profile and HbA1c, and normalization of blood glucose are among the undeniable benefits of ketogenic and carnivore diets [14]. Although minimally studied in children, there is some data on the long-term effects of the ketogenic diet in pediatric patients with epilepsy [5]. This data should theoretically be transferable to overweight and diabetic children being managed with a ketogenic or ketogenic-like diet. When properly implemented, studies on carnivore and ketogenic-like diets suggest several long-term benefits for patients, including increased energy expenditure, improved sleep quality, reduced respiratory quotient, better management of hyperlipidemia, weight loss, improved HbA1c levels, normalized blood glucose, and enhanced control of comorbidities, leading to a lower risk of metabolic syndrome and certain cancers [5]. These benefits are also reported in Figure 1.

Although the benefits of these diets are tremendous, skeptics have expressed concerns about both ketogenic and carnivore diets. The main concern regarding the carnivore diet is that the participants are unable to obtain essential nutrients found only in plant sources. The long-term consequences of this diet are also unknown, and in some cases elevated urinary calcium-creatinine ratios have been detected (with no hypercalcemia) in one out of 20 patients who were on a ketogenic diet, and this could possibly lead to kidney stone formation [12]. While research indicates that following a ketogenic diet for six to 12 months is generally safe, the long-term effects of prolonged adherence remain unclear [5]. In a four-month study of a ketogenic diet in young adult rats, multiple cerebral changes were observed. Key findings included increases in metabolites and antioxidants, along with alterations in structure and intracerebral connectivity. Specifically, glutamate and glutamine levels rose, which together increase the nervous system's excitatory responses, indicating the ketogenic diet’s ability to provide more energy than a normal diet. Additionally, the ketogenic diet increased glutathione, an important free radical scavenger responsible for reducing the damage caused by oxidation. The study also revealed an increase in N-acetylaspartate (NAA) and N-acetylaspartylglutamate (NAAG) in the hippocampal formation. While the full role of these molecules remains unclear, they are believed to contribute to myelin and mitochondrial formation. This increase likely explains the study’s findings of increased myelinated fibers in the striatum as well as enhanced cerebral connections between the striatum and the internal capsule with subsequent increase in the volume of the pons and hippocampal formation, all of which are interconnected and belong to the same functional subsystem [13].

While glutamine plays a role in supporting the brain’s energy expenditure, it can inadvertently fuel processes the body would prefer to restrict, such as cancer growth. Research indicates that glutamine is a primary mitochondrial substrate for cancer cells, particularly in pancreatic ductal adenocarcinoma (PDAC), helping these cells maintain their energy. Current studies are exploring glutamine deprivation and the knockdown of enzymes in the tricarboxylic acid (TCA) cycle as potential therapeutic approaches for targeting these specific tumors [15,16]. Contrary to the idea that plants are required to fulfill specific nutrient requirements, all essential nutrients can be found in foods sourced from animals. In fact, nutrients from animal sources are more bioavailable when compared to those from plant sources [6]. It is suggested that the unusually large and complex human brain evolved, in part, due to our ancestors' diets being primarily composed of animal-sourced foods. This dietary shift is also believed to have contributed to the higher stomach acidity observed in humans compared to chimpanzees [17].

The human body has evolved to rely on animal-sourced foods as a dietary foundation. However, modern Western diets have shifted toward being predominantly carbohydrate-based. While data increasingly support the notion that humans are biologically suited to a carnivorous or animal-protein-heavy diet, current international guidelines for managing pediatric obesity continue to emphasize reduced protein intake and increased vegetable consumption [1]. A shift toward higher-protein dietary recommendations could potentially reduce the prevalence of pediatric obesity. This is because protein requires more energy to metabolize than carbohydrates and promotes greater satiety, leading to a lower overall calorie intake. That said, concerns around the ketogenic and carnivore diets are valid, particularly due to the limited long-term research in pediatric populations. One study, however, showed that sustained weight loss without rebound weight gain was achieved using two short cycles of a ketogenic diet, interspersed with periods of maintenance on a Mediterranean diet [5]. Using this strategy could help ease the concerns surrounding both ketogenic and carnivore diets.

Given the lack of long-term research surrounding these diets, there are several directions for further investigation. It is suggested that nutritionists be included in future research, and informative yet community-friendly material be provided to parents of children at risk or to those suffering from pediatric obesity and diabetes. These materials can include, but should not be limited to, pamphlets with listed meal plans to aid parents in the preparation of their child’s diet.

## Conclusions

While the carnivore diet has been minimally studied in pediatric populations, ketogenic diets have been extensively researched and documented. The results of this study suggest that humans can get all the required nutrients when they are on a strict carnivore diet, and due to the likeness of the ketogenic diet, we can infer there will be minimal side effects and long-term detrimental consequences of implementing a temporary carnivore-like diet to manage pediatric obesity. Pediatric obesity is multifactorial, and adopting a holistic approach to prevention and treatment is one of the most valuable aspects of patient care in these cases. Osteopathic principles and practices can be used to determine the sources and comorbidities common in children suffering from obesity and alleviate potential risk factors early in development. While there is no single cure for pediatric obesity, a combination of proper nutrition, regular physical activity, and a holistic, patient-centric approach can significantly reduce the risk of its development.
